# Multidisciplinary Expert Perspectives on a Conceptual Autonomous Mini Surgical Robot for Gynecological Procedures: An Exploratory Qualitative Study

**DOI:** 10.3390/healthcare14152306

**Published:** 2026-07-31

**Authors:** Özen İnam, Yahya Kahvecioğlu, Samet Okay

**Affiliations:** 1 Department of Midwifery, School of Health Sciences, Maltepe University, 34857 İstanbul, Türkiye; 2 First and Emergency Aid Program, Department of Medical Services and Techniques, Maltepe University, 34857 İstanbul, Türkiye; yahyakahvecioglu@maltepe.edu.tr; 3 Anesthesia Program, Department of Medical Services and Techniques, Maltepe University, 34857 İstanbul, Türkiye; sametokay@maltepe.edu.tr

**Keywords:** healthcare technology implementation, autonomous surgical robot, gynecological surgery, artificial intelligence, qualitative research, technology adoption, women’s health, patient safety

## Abstract

**Highlights:**

**What are the main findings?**

- Multidisciplinary experts identified physician oversight, safety validation, workflow integration, and regulatory preparedness as essential prerequisites for implementing autonomous mini surgical robots in gynecological practice.
- Healthcare professionals and engineers suggest complementary perspectives, with clinicians prioritizing patient safety and usability, while engineers emphasized technological optimization and artificial intelligence integration.

**What are the implications of the main findings?**

- Early multidisciplinary stakeholder evaluation may facilitate the responsible implementation and adoption of autonomous surgical robotics in hospital settings.
- The findings provide practical guidance for clinicians, engineers, healthcare organizations, and technology developers planning the implementation of next-generation robotic systems in gynecological care.

**Abstract:**

**Background/Objectives:** Successful implementation of autonomous surgical technologies requires not only engineering innovation but also early evaluation of clinical usability, patient safety, workflow integration, and stakeholder acceptance. Although autonomous robotic systems are expected to transform minimally invasive surgery, evidence regarding their implementation in gynecological practice remains limited. This study explored multidisciplinary expert perspectives on the clinical implementation, safety, usability, workflow integration, and future adoption of an autonomous mini surgical robot concept designed for intrauterine polyp resection and tubal ligation. **Methods:** A qualitative study was conducted with 10 experts (3 gynecologists, 2 anesthesiologists, 2 women’s health nurses, and 3 engineers). Participants evaluated the proposed concept through open-ended questionnaires and semi-structured interviews. Data were analyzed using Braun and Clarke’s thematic analysis. **Results:** Six implementation-oriented themes were identified: clinical value, patient safety and human oversight, technology readiness, clinical workflow integration, future adoption and scalability, and ethical governance. Participants considered the proposed concept to have the potential to improve procedural precision and standardization while emphasizing that successful implementation would require physician supervision, rigorous safety validation, integration into existing clinical workflows, multidisciplinary collaboration, professional training, and appropriate ethical and regulatory oversight. Clinicians primarily emphasized patient safety and usability, whereas engineers focused on technological optimization and AI integration. **Conclusions:** This exploratory qualitative study provides preliminary insights into multidisciplinary expert perspectives on the future implementation of autonomous mini surgical robots in gynecology. The findings may help inform future technology development, implementation planning, and subsequent prototype-based clinical research.

## 1. Introduction

Artificial intelligence (AI), digital health technologies, and robotic systems are transforming healthcare delivery by improving procedural precision, patient safety, clinical efficiency, and decision support. As healthcare organizations increasingly adopt innovative technologies, successful implementation depends not only on engineering advances but also on their integration into clinical workflows, acceptance by healthcare professionals, compliance with regulatory requirements, and alignment with patient-centered care [1,2]. Consequently, the evaluation of emerging healthcare technologies has become an essential component of implementation planning before routine clinical adoption [1,3]. Among these technological advances, robotic-assisted surgery has substantially transformed minimally invasive surgical practice over the past two decades [3,4].

Robotic surgical platforms were developed to overcome several limitations of conventional laparoscopy by enhancing dexterity, visualization, precision, and surgeon ergonomics [2,4,5]. These technological improvements have enabled surgeons to perform increasingly complex procedures with greater accuracy and consistency, contributing to the widespread integration of robotic surgery across multiple surgical specialties, including gynecology [2,5]. Minimally invasive surgery has become the preferred approach for many gynecological procedures because it is associated with reduced blood loss, less postoperative pain, shorter hospitalization, faster recovery, and improved patient satisfaction [2,6]. Robotic-assisted gynecological surgery has provided additional advantages, particularly for procedures performed within anatomically confined spaces that require delicate tissue manipulation and high procedural precision. Nevertheless, currently available robotic platforms remain relatively large, costly, infrastructure-dependent, and reliant on continuous surgeon control, limiting their widespread implementation across different healthcare settings [2,7].

Recent advances in robotics, artificial intelligence, microelectronics, sensor technologies, and battery systems have accelerated the development of miniaturized and microrobotic surgical platforms capable of operating within confined anatomical environments [2]. These technologies have attracted considerable interest because of their potential to perform highly targeted interventions while minimizing tissue trauma and preserving surrounding anatomical structures [8,9]. Emerging applications include targeted drug delivery, microscale diagnostics, tissue sampling, and minimally invasive therapeutic procedures [10,11]. As technological capabilities continue to evolve, increasing attention has shifted from technical feasibility toward the future clinical implementation of autonomous robotic systems [9].

Recent advances in miniaturized robotic technologies have increasingly shifted attention from engineering feasibility toward clinical translation and implementation readiness. Ceylan et al. (2026) emphasized that successful translation of medical microrobots requires not only technological maturity but also alignment with unmet clinical needs, integration into existing clinical workflows, multidisciplinary collaboration, and demonstration of clinical value [12]. Complementing this translational perspective, recent clinical experience with next-generation robotic platforms, particularly the da Vinci SP system, suggests the ongoing transition toward less invasive and more compact robotic technologies in gynecological surgery [13,14,15]. Miniaturized robotic technologies may offer particularly important opportunities for women’s healthcare. Many gynecological procedures are performed within narrow anatomical cavities where procedural precision, tissue preservation, and patient comfort are essential for achieving optimal clinical outcomes [9,16].

Beyond their direct clinical benefits, mini robotic systems may improve procedural standardization, reduce operator-dependent variability, and support more consistent delivery of minimally invasive care through integration with advanced imaging systems and artificial intelligence [1,3,6]. Such developments have the potential to facilitate broader implementation of innovative surgical technologies across different hospital settings. An important evolution in surgical robotics is the gradual transition from surgeon-controlled systems toward increasingly autonomous platforms [3]. Although fully autonomous surgery remains a long-term objective, advances in machine learning, computer vision, sensor fusion, and real-time navigation technologies have enabled robotic systems to perform selected procedural tasks with varying degrees of autonomy [1,3]. These developments suggest that future robotic systems may increasingly function as intelligent clinical partners capable of supporting standardized procedural tasks under appropriate physician supervision. In the present study, autonomy refers to supervised autonomy, whereby the proposed robotic system performs predefined procedural tasks autonomously under continuous physician supervision, while the physician retains authority to approve, interrupt, redirect, or terminate the procedure whenever necessary.

Among potential gynecological applications, intrauterine polyp resection and tubal ligation represent particularly suitable clinical scenarios for evaluating autonomous mini robotic concepts because both procedures involve relatively standardized workflows performed within confined anatomical environments [1,9]. These characteristics provide an appropriate framework for evaluating autonomous navigation, task execution, physician oversight, workflow integration, patient safety, and implementation requirements. Furthermore, the standardized nature of these procedures allows implementation-related factors identified in this study to inform the future development of other minimally invasive gynecological robotic applications.

Implementation science suggests that successful integration of innovative healthcare technologies depends not only on technical performance but also on organizational readiness, workflow integration, professional acceptance, multidisciplinary collaboration, and regulatory considerations. While these implementation dimensions have been widely discussed for digital health technologies, comparatively limited attention has been given to how healthcare professionals perceive the future implementation of increasingly miniaturized and autonomous robotic systems in gynecological surgery despite the growing clinical experience with single-port robotic platforms [13,15].

Despite the rapid advancement of robotic and microrobotic technologies, existing research has predominantly focused on engineering design, technical feasibility, and experimental validation [1,9]. Comparatively little attention has been given to how healthcare professionals perceive the future clinical implementation, workflow integration, organizational adoption, safety, and ethical acceptability of autonomous surgical technologies [13,14,15]. Understanding these perspectives is essential because successful implementation of healthcare technologies depends not only on technical performance but also on acceptance by clinicians, nurses, engineers, and other key stakeholders involved in healthcare delivery [1,17]. Although this study was not explicitly designed around a single implementation framework, concepts from implementation science—including organizational readiness, workflow integration, professional acceptance, and multidisciplinary collaboration—guided the development of the interview topics and informed the interpretation of the findings.

The aim of this exploratory qualitative study was to explore multidisciplinary expert perspectives regarding the clinical implementation, patient safety, usability, workflow integration, and future adoption of an autonomous mini surgical robot concept designed for intrauterine polyp resection and tubal ligation. By integrating the perspectives of gynecologists, anesthesiologists, women’s health nurses, and engineers, this exploratory qualitative study provides preliminary multidisciplinary insights into the clinical value, implementation requirements, ethical considerations, and organizational factors relevant to the future development of autonomous surgical robotics in gynecological care.

The remainder of this paper is organized as follows. Section 2 describes the methodology, Section 3 presents the findings, Section 4 discusses the results in relation to the existing literature, and Section 5 concludes the study.

## 2. Materials and Methods

### 2.1. Study Design

This qualitative study was designed to explore multidisciplinary expert perspectives on the clinical implementation, adoption, and future integration of an autonomous mini surgical robot concept developed for minimally invasive gynecological procedures. A qualitative approach was considered appropriate because the study sought to obtain an in-depth understanding of expert perceptions regarding clinical applicability, patient safety, workflow integration, usability, technical feasibility, and organizational factors that may influence the implementation of an emerging healthcare technology. Data were collected through a combination of open-ended questionnaires and semi-structured interviews.

### 2.2. Participants and Data Collection

A purposive sampling strategy was used to recruit experts from gynecology, anesthesiology, women’s health nursing, and engineering to ensure multidisciplinary evaluation of the proposed autonomous mini surgical robot concept. The final sample consisted of three gynecologists, two anesthesiologists, two women’s health nurses, and three engineers. Participants were purposively recruited from multiple hospitals, universities, and engineering organizations to ensure multidisciplinary perspectives and variation in professional experience. Potential participants were identified through the professional networks of the research team and invited by e-mail or direct personal contact according to the predefined eligibility criteria. To be eligible for participation, experts were required to have at least five years of professional experience in their respective fields and to voluntarily agree to participate in the study. In addition, gynecologists were required to have active experience in gynecological ultrasonography and operative gynecology, including routine performance of gynecological surgical procedures. This criterion was established to ensure that participants possessed sufficient practical expertise to evaluate the proposed robotic concept from clinical and engineering perspectives.

The professional characteristics, years of professional experience, academic positions, and relevant areas of expertise of the participants are summarized in Table 1.

The sample size was determined according to the principle of data saturation, a widely accepted criterion in qualitative research [18,19]. Data collection continued until no substantially new themes or concepts emerged from participant responses.

Before each interview, all participants were presented with standardized visual schematics and a structured conceptual description of the proposed autonomous mini surgical robot. The presentation included its intended clinical applications, technical specifications, operational workflow, physician-supervised semi-autonomous operation, navigation strategy, imaging system, surgical modules, safety mechanisms, and representative procedural sequence. Participants were informed that the platform represented a conceptual design rather than a functional prototype. They were encouraged to ask clarification questions before the interview, and additional explanations were provided when necessary without modifying the underlying concept or introducing additional design features. This approach ensured that all participants evaluated the same technological concept under consistent conditions. Following this standardized briefing and discussion, semi-structured interviews were conducted either face-to-face or online. The duration of the interviews varied depending on the extent of participants’ responses and the need for follow-up probing questions, with an average duration of approximately 20 min. To encourage open discussion and maximize participant comfort, responses were documented in written form during the interviews rather than audio-recorded. When necessary, additional probing questions were asked to clarify responses and obtain more detailed explanations.

### 2.3. Data Collection Instruments

Data were collected using two complementary instruments. First, participants completed a brief demographic form including age, gender, profession, and academic/professional title, followed by an open-ended questionnaire to obtain their initial perceptions regarding the robot’s design characteristics, anticipated clinical value, patient safety, usability, implementation feasibility, and potential role within gynecological practice. The questionnaire responses served as an initial exploration of participants’ perspectives and informed the subsequent semi-structured interviews.

Second, a semi-structured interview guide was used to clarify, expand, and further explore the issues identified in the questionnaire responses. The interview guide consisted of the following core questions:

1. What is your overall evaluation of the proposed autonomous mini surgical robot design?
2. How do you evaluate the clinical applicability of this system?
3. What are your opinions regarding the technical and functional characteristics of the proposed design?
4. What potential impact do you think this technology may have on your professional role and routine clinical practice?
5. What suggestions would you make to improve the proposed robotic system?
6. How would you describe your overall attitude toward the future clinical adoption of this type of technology?

When appropriate, follow-up probing questions were used to clarify participants’ responses and to explore emerging topics in greater depth.

### 2.4. Description of the Autonomous Mini Surgical Robot Concept

**Figure 1:**
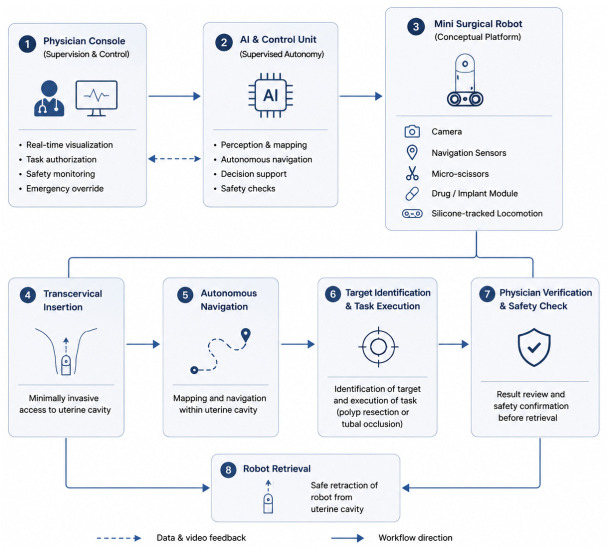
Conceptual architecture and representative workflow of the proposed autonomous mini surgical robot.

The autonomous mini surgical robot evaluated in this study was designed as a conceptual microrobotic platform intended for selected intrauterine procedures. The proposed system incorporates autonomous navigation based on three-dimensional uterine mapping generated from preoperative magnetic resonance imaging or ultrasonographic data. These preoperative imaging data are conceptually intended to identify the target anatomy and generate a predefined navigation pathway before the procedure. During the intervention, the integrated micro-camera provides real-time visual feedback to verify the navigation pathway, compensate for minor anatomical or patient positional variations, and support continuous physician supervision throughout the procedure. The conceptual design includes a soft silicone-based tracked locomotion mechanism for intrauterine navigation, a micro-camera with integrated LED illumination, micro-scissor and micro-clip mechanisms for tissue manipulation, and a targeted drug-delivery compartment. Conceptually, the robot is intended to be introduced into the uterine cavity through the cervical canal under physician supervision. For intrauterine polyp resection, the robot is designed to autonomously navigate to the target lesion within the uterine cavity. For the tubal ligation scenario, the robot is conceptually intended to navigate toward the uterine cornua and enter the proximal intramural segment of the fallopian tube, where a biocompatible micro-occlusion implant would be deployed to achieve tubal occlusion. This proposed mechanism represents a conceptual design intended to facilitate expert evaluation and has not yet undergone prototype development or experimental validation. The conceptual architecture and representative procedural workflow of the proposed robotic platform, corresponding to the standardized concept presented to all participants before the interviews, are illustrated in Figure 1 to facilitate a consistent understanding of the system evaluated throughout the study.

As illustrated in Figure 1, the proposed system is conceptualized as a physician-supervised semi-autonomous surgical platform in which autonomous navigation and predefined procedural tasks are continuously monitored by the supervising physician. Autonomous functions include intrauterine navigation, anatomical localization, target identification, route planning, and execution of predefined procedural tasks. However, all irreversible surgical interventions, including tissue resection and deployment of the micro-occlusion implant, would require explicit physician approval before execution. Throughout the procedure, the supervising physician would retain full authority to monitor the robot in real time and, through a secure wireless communication interface, pause, redirect, abort, or retrieve the robot whenever necessary. In the event of unexpected anatomical findings, technical malfunction, or any safety concern, the autonomous workflow would be immediately interrupted, allowing physician intervention or conversion to a conventional surgical approach if required.

### 2.5. Rationale for the Selected Clinical Scenarios

Intrauterine polyp resection and tubal ligation were selected as representative clinical scenarios because both procedures are performed within relatively confined anatomical spaces, require high procedural precision, and generally follow standardized operative workflows. Tubal ligation was specifically selected because it is an elective procedure with well-defined and standardized operative steps, making it an appropriate conceptual model for evaluating autonomous robotic applications. In addition, a minimally invasive transcervical approach has the potential to reduce the need for conventional surgery, hospitalization, and operating room resources, while potentially improving patient acceptance of permanent contraception. These characteristics make both procedures suitable models for evaluating not only the technical capabilities of miniaturized robotic systems but also the practical challenges associated with clinical implementation, workflow integration, patient safety, and future technology adoption. These clinical scenarios were included as conceptual use cases to support multidisciplinary evaluation of the proposed technology rather than to describe an established or clinically validated surgical technique.

### 2.6. Role of the Robot Concept in the Evaluation

The proposed robotic platform served as the central evaluation object throughout the study. Rather than assessing a commercially available device, participants evaluated an early-stage conceptual design from both clinical and engineering perspectives. They were asked to identify perceived strengths, implementation opportunities, safety concerns, workflow implications, and future development priorities. Presenting the concept at an early stage enabled stakeholders to provide recommendations that may support subsequent technology refinement and implementation planning.

### 2.7. Data Analysis

The qualitative data were analyzed using Braun and Clarke’s six-phase thematic analysis framework [20]. The researchers first familiarized themselves with the data by repeatedly reviewing the interview notes. Meaningful statements were then identified and coded using an inductive approach. Conceptually related codes were grouped into preliminary categories, which were iteratively reviewed, refined, and organized into overarching themes. Finally, the themes were defined, named, and agreed upon through discussion among the research team.

Two researchers independently performed the coding process and compared their findings throughout the analysis. Discrepancies were resolved through discussion until consensus was achieved. To minimize individual interpretation bias, themes were developed directly from participants’ responses through iterative comparison and collaborative discussion rather than researcher assumptions. The final themes were considered to represent the most prominent implementation-related patterns emerging from the dataset. The research team included healthcare professionals and engineers. To minimize the potential influence of researchers’ professional backgrounds on data interpretation, themes were developed inductively from participants’ responses, and coding decisions were reached through independent coding and consensus-based discussion. Because the primary objective of this exploratory pilot study was to obtain multidisciplinary perspectives on a single conceptual robotic platform rather than to compare views across professional groups, the analysis focused on identifying themes across the overall multidisciplinary dataset. Accordingly, the findings should not be interpreted as demonstrating thematic saturation within each professional subgroup.

As this study was designed as a pilot qualitative investigation, the primary objective was to obtain initial multidisciplinary expert perspectives on the proposed robotic concept rather than to develop an exhaustive theoretical framework. Data collection continued until no substantially new themes or perspectives emerged during the analysis, suggesting sufficient thematic richness across the overall multidisciplinary dataset. However, given the limited number of participants within each professional group, thematic saturation cannot be assumed at the subgroup level. During the later interviews, participants’ responses became largely repetitive and frequently reflected previously identified themes. This convergence was considered expected because participants evaluated the same conceptual robotic design rather than a functioning prototype, limiting variation beyond the proposed features of the system.

### 2.8. Ethical Considerations

Ethical approval for this study was obtained from the Ethics Committee of Maltepe University (Approval No: 2026/09-14). All participants received detailed information regarding the purpose of the study and provided written informed consent before participation. Confidentiality and anonymity were maintained throughout data collection, analysis, and reporting. ChatGPT (OpenAI; GPT-5.5-mini) was used during manuscript preparation to assist with English translation, language editing, and the preparation of conceptual graphical illustrations. It was not used in the design of the study, participant recruitment, data collection, data analysis, interpretation of the findings, or generation of the scientific conclusions. All AI-assisted outputs were carefully reviewed, verified, and edited by the authors, who take full responsibility for the accuracy and integrity of the manuscript.

## 3. Results

Thematic analysis of interviews conducted with ten multidisciplinary experts identified six major themes describing perceptions of the proposed autonomous mini surgical robot for gynecological procedures. These themes reflected participants’ views regarding clinical value, patient safety, technical readiness, workflow integration, implementation readiness, and ethical considerations associated with the proposed technology. Table 2 summarizes the identified themes, their descriptions, representative participant statements reconstructed from contemporaneous written interview notes, and the primary professional perspectives derived from the thematic analysis.

**Figure 2:**
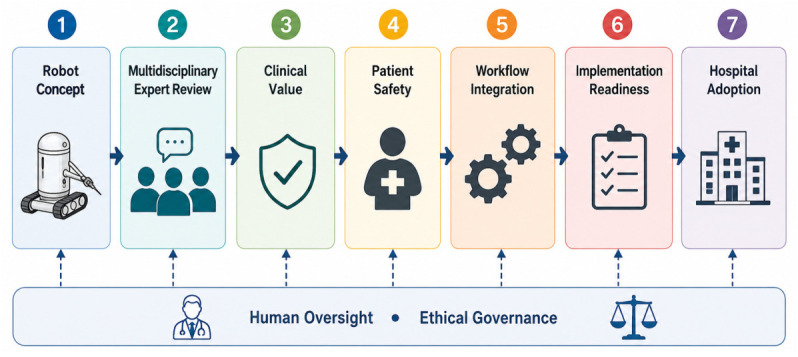
Conceptual implementation framework for the proposed autonomous mini surgical robot. The numbered components (1–7) represent the key themes identified through thematic analysis: (1) Robot Concept, (2) Multidisciplinary Expert Review, (3) Clinical Value, (4) Patient Safety, (5) Workflow Integration, (6) Implementation Readiness, and (7) Hospital Adoption. Dashed arrows indicate the supporting role of human oversight and ethical governance across all implementation components.

Figure 2 presents the conceptual implementation framework of the proposed autonomous mini surgical robot. The framework illustrates the interrelationships among the themes identified through thematic analysis and summarizes the implementation-oriented perspectives that emerged from participants’ evaluations, including clinical value, patient safety, workflow integration, implementation readiness, and hospital adoption.

### 3.1. Perceived Clinical Benefits

Participants commonly described the proposed mini robotic system as a promising innovation that could support minimally invasive gynecological procedures. The most frequently anticipated benefits included reduced procedural trauma, improved surgical precision, shorter procedure duration, enhanced procedural standardization, and increased patient comfort. Although clinicians primarily focused on patient outcomes and quality of care, engineers more often discussed the system’s potential to improve technical performance and reduce operator-dependent variability.

A gynecologist stated:

*“Especially in routine and short gynecological procedures, such systems may reduce procedural difficulty and increase precision.”*

(P2, Gynecologist)

A women’s health nurse emphasized patient comfort:

*“Women may feel less anxious during minimally invasive procedures supported by robotic systems.”*

(P5, Women’s Health Nurse)

An engineering participant focused on technological efficiency:

*“The mini robotic platform may standardize repetitive procedures and reduce operator-dependent variability.”*

(P8, Mechanical Engineer)

Overall, participants regarded the proposed system as a promising technology with potential applications in minimally invasive gynecological procedures. Taken together, these quotations suggest that participants perceived the proposed robotic platform as having the potential to improve procedural precision, reduce invasiveness, and enhance patient comfort, while emphasizing that these anticipated benefits require future experimental and clinical validation.

### 3.2. Concerns Regarding Autonomy and Safety

Concerns regarding patient safety, autonomous decision-making, and the continued need for physician oversight emerged frequently across all professional groups. Healthcare professionals emphasized the importance of maintaining human supervision throughout robotic procedures, whereas engineers focused on technological safeguards that could reduce procedural risks.

One gynecologist stated:

*“A fully autonomous system may create risks in unexpected complications. Physician supervision should always remain active.”*

(P2, Gynecologist)

Another gynecologist raised concerns regarding tissue injury:

*“The system must be highly sensitive while navigating within the uterine cavity. Even minor uncontrolled movements may cause injury.”*

(P3, Gynecologist)

A women’s health nurse emphasized emotional safety:

*“Patients may initially feel anxious about robotic procedures. The presence of healthcare professionals would still be necessary to provide emotional reassurance.”*

(P5, Women’s Health Nurse)

An engineering participant approached safety from a technical perspective:

*“Safety algorithms and real-time feedback systems could significantly minimize procedural risks.”*

(P8, Electrical and Electronics Engineer)

Although perspectives differed according to professional background, participants frequently emphasized that autonomous robotic systems should function under continuous physician supervision and incorporate robust safety mechanisms before clinical implementation. These findings suggest that physician supervision was frequently regarded as an essential prerequisite for the safe implementation of autonomous robotic technologies.

### 3.3. Technical Functionality and Technology Readiness

Participants evaluated the proposed autonomous mini surgical robot with respect to its technical capabilities, maneuverability, navigation accuracy, imaging support, and overall system performance. Engineering participants primarily emphasized technological optimization, autonomous navigation, and integration of advanced imaging systems, whereas healthcare professionals focused on practical usability and compatibility with routine gynecological practice. Overall, many participants suggested that further technological refinement would be necessary before successful clinical implementation.

One engineering participant emphasized the importance of navigation precision:

*“The robot should have highly precise movement capability within the uterine cavity. Even millimetric deviations may affect procedural safety.”*

(P8, Mechanical Engineer)

Another engineering participant discussed the value of imaging integration:

*“Real-time imaging systems and sensor-supported navigation would significantly improve the success of the procedure.”*

(P9, Electrical and Electronics Engineer)

A gynecologist emphasized the importance of usability during routine clinical practice:

*“The system should be simple and practical enough to be used comfortably during routine gynecological procedures.”*

(P2, Gynecologist)

A women’s health nurse focused on compatibility with existing clinical workflows:

*“The device should not complicate the clinical process. It should support healthcare professionals rather than increasing workload.”*

(P5, Women’s Health Nurse)

Another engineering participant suggested the future integration of artificial intelligence:

*“Artificial intelligence-supported motion analysis and automatic positioning systems may improve procedural precision in the future.”*

(P10, Computer Engineer)

Overall, participants recognized the technological potential of the proposed concept while emphasizing that prototype development, technical optimization, and experimental validation remain important prerequisites for future clinical implementation.

### 3.4. Effects on Professional Roles and Clinical Workflow

Participants discussed the potential influence of autonomous mini robotic systems on professional responsibilities, clinical workflow, and healthcare delivery. While several participants believed that robotic assistance could improve procedural efficiency and reduce repetitive workload, they frequently emphasized that autonomous systems should function as supportive technologies rather than replacements for healthcare professionals. Human oversight and patient-centered care were considered important components of future clinical implementation.

One gynecologist stated:

*“Such systems may reduce the duration of routine procedures and provide more standardized interventions.”*

(P1, Gynecologist)

Another gynecologist emphasized the continuing importance of physician supervision:

*“Even if robotic systems are integrated into practice, the physician should remain the primary decision-maker throughout the procedure.”*

(P3, Gynecologist)

Women’s health nurses particularly discussed the importance of communication and emotional support:

*“Technology may facilitate procedures, but patients still need emotional reassurance and communication from healthcare professionals.”*

(P4, Women’s Health Nurse)

Another nurse emphasized maintaining the human aspect of care:

*“The robotic system should support healthcare professionals rather than replacing the human aspect of care.”*

(P6, Women’s Health Nurse)

An engineering participant focused on workflow optimization:

*“Automation may reduce repetitive manual tasks and improve procedural consistency within clinical settings.”*

(P9, Electrical and Electronics Engineer)

Although participants acknowledged the potential of robotic technologies to improve efficiency and standardization, they frequently viewed the proposed system as a complementary technology intended to support, rather than replace, healthcare professionals.

### 3.5. Future Recommendations and Expansion Potential

Participants provided several recommendations regarding the future development of the proposed autonomous mini surgical robot and its potential implementation across a broader range of gynecological procedures. The recommendations primarily focused on improving imaging capabilities, strengthening artificial intelligence integration, enhancing procedural safety, expanding clinical applications, and supporting successful implementation through professional training and validation.

One gynecologist suggested extending the system to additional minimally invasive procedures:

*“The system could be expanded for use in hysteroscopic procedures and other minimally invasive gynecological interventions in the future.”*

(P2, Gynecologist)

Another gynecologist emphasized the importance of comprehensive validation before clinical use:

*“Before clinical implementation, the system should undergo extensive testing and validation to minimize procedural risks.”*

(P3, Gynecologist)

A women’s health nurse emphasized the importance of patient-centered design:

*“The design should prioritize patient comfort and privacy, especially during sensitive gynecological procedures.”*

(P5, Women’s Health Nurse)

Another nurse emphasized the need for professional preparation before implementation:

*“Healthcare professionals should receive adequate training before integrating such robotic systems into clinical practice.”*

(P6, Women’s Health Nurse)

An engineering participant focused on future technological development:

*“Artificial intelligence-supported navigation and automated motion correction systems may significantly improve procedural accuracy in future versions.”*

(P8, Mechanical Engineer)

Another engineering participant recommended integration with advanced imaging technologies:

*“Combining the robotic platform with real-time imaging and sensor technologies could improve both safety and precision.”*

(P10, Computer Engineer)

Overall, many participants viewed the proposed system as a promising healthcare innovation with potential for future development. Their recommendations primarily focused on technological refinement, comprehensive validation, professional training, and gradual integration into clinical practice.

### 3.6. Ethical and Legal Considerations

Ethical and legal considerations emerged as another major theme during the interviews. Participants discussed issues related to patient safety, professional accountability, data security, physician responsibility, and regulatory oversight. Across professional groups, many participants considered ethical governance to be important for the responsible implementation of autonomous surgical robotics in clinical practice.

One gynecologist stated:

*“In the event of a complication, determining responsibility may become a major ethical and legal issue.”*

(P1, Gynecologist)

Another gynecologist emphasized the importance of maintaining physician authority:

*“A robotic system should never independently make critical clinical decisions without physician approval.”*

(P3, Gynecologist)

A women’s health nurse emphasized patient trust:

*“Patients may hesitate to trust fully autonomous systems during intimate gynecological procedures.”*

(P4, Women’s Health Nurse)

Another nurse focused on privacy and confidentiality:

*“Protection of patient data and procedural confidentiality would be essential for widespread acceptance of such technologies.”*

(P6, Women’s Health Nurse)

An engineering participant addressed ethical risks from a technical perspective:

*“Ethical risks could be reduced through advanced safety protocols and controlled authorization systems.”*

(P8, Mechanical Engineer)

Another engineering participant emphasized the need for regulatory standards:

*“Clear legal regulations and technical standards would be necessary before these systems could be integrated into routine clinical practice.”*

(P9, Electrical and Electronics Engineer)

**Figure 3:**
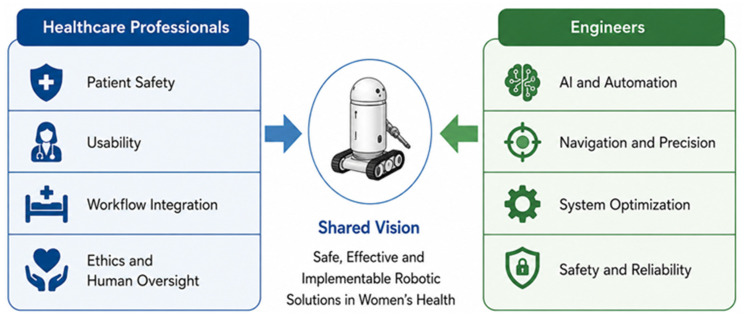
Comparison of multidisciplinary perspectives on the future implementation of the proposed autonomous mini surgical robot.

Figure 3 summarizes the multidisciplinary perspectives identified through thematic analysis, illustrating both the shared vision and the discipline-specific priorities of healthcare professionals and engineers regarding the future implementation of the proposed autonomous mini surgical robot.

As illustrated in Figure 3, participants from both professional groups expressed generally consistent support for the proposed robotic concept while emphasizing different priorities according to their disciplinary backgrounds. Healthcare professionals primarily focused on patient safety, clinical usability, workflow integration, and physician oversight, whereas engineers emphasized artificial intelligence, autonomous navigation, system optimization, and technical reliability. Despite these differences, both groups shared the common vision that prototype development, rigorous validation, appropriate regulatory frameworks, and continuous physician supervision would be essential prerequisites before clinical implementation.

## 4. Discussion

This study explored multidisciplinary expert perspectives on the implementation potential of a proposed autonomous mini surgical robot designed for intrauterine polyp resection and tubal ligation. Overall, participants recognized the concept as a promising healthcare innovation with the potential to improve procedural precision, reduce tissue trauma, enhance patient comfort, and support standardized minimally invasive gynecological procedures. At the same time, participants frequently emphasized that successful implementation would depend on maintaining patient safety, physician oversight, ethical governance, and appropriate regulatory frameworks. These findings suggest that the future adoption of autonomous surgical robotics in gynecology will be influenced not only by technological capability but also by clinical acceptance, organizational readiness, and responsible implementation strategies. As the proposed robot represents an early-stage conceptual design without prototype or experimental validation, these findings should be interpreted as expert perceptions regarding its potential implementation rather than evidence of clinical effectiveness.

One of the principal findings of this study was that participants perceived mini robotic systems as having the potential to improve procedural precision and consistency during minimally invasive gynecological interventions. Experts particularly emphasized the advantages of operating within the confined anatomical environment of the uterine cavity, where precise navigation and controlled tissue manipulation are critical. These findings are consistent with previous studies demonstrating that robotic-assisted surgery improves dexterity, visualization, motion accuracy, and procedural standardization while reducing operator-dependent variability [21]. Similar benefits have also been reported in gynecological robotic surgery, where enhanced precision has been associated with reduced tissue trauma and improved postoperative recovery [1,22].

Accordingly, participants anticipated that a future autonomous mini robotic platform with similar capabilities may provide comparable advantages, although such expectations require confirmation through prototype development, experimental testing, and clinical validation. However, previous studies have also noted that the implementation of robotic surgical technologies may be constrained by factors such as acquisition costs, unequal access to advanced technologies, infrastructure requirements, and the need for specialized training. These considerations suggest that successful implementation depends not only on technological innovation but also on healthcare system capacity and organizational preparedness.

Patient safety emerged as another central finding. Although participants acknowledged the potential benefits of increasing surgical autonomy, they frequently emphasized that autonomous robotic systems should operate under continuous physician supervision. Concerns regarding unexpected complications, tissue injury, and maintenance of clinical control were frequently expressed by gynecologists and women’s health nurses. These findings are consistent with previous reports identifying trust, safety, accountability, and human oversight as major determinants of acceptance for autonomous surgical systems [2]. Likewise, Granados et al. (2026) argued that increasing levels of surgical autonomy should be accompanied by robust physician oversight and clearly defined clinical responsibilities [23].

The multidisciplinary composition of the participant group provided valuable insights into different implementation priorities. Healthcare professionals primarily emphasized patient safety, emotional reassurance, clinical usability, and workflow compatibility, whereas engineers focused on technological feasibility, artificial intelligence integration, navigation accuracy, and system optimization. Similar differences between clinical and engineering perspectives have been reported in previous studies evaluating emerging healthcare technologies, where clinicians generally prioritize patient-centered outcomes while engineers focus on technical performance and system development [24]. Rather than representing conflicting viewpoints, these complementary perspectives underscore the importance of multidisciplinary collaboration throughout the design, evaluation, and implementation of future autonomous surgical systems.

Ethical and legal issues represented another important dimension of technology implementation. Participants expressed concerns regarding professional accountability, patient privacy, data security, physician responsibility, and autonomous decision-making. These findings reflect ongoing discussions surrounding artificial intelligence and autonomous healthcare technologies, particularly regarding transparency, responsibility, and regulatory oversight [1]. Previous studies have similarly emphasized that clear regulatory frameworks, governance mechanisms, and ethical standards are fundamental prerequisites for the responsible implementation of autonomous robotic systems in healthcare [23]. The present findings reinforce the view that ethical governance should evolve alongside technological innovation to support sustainable clinical adoption. At the same time, developing appropriate ethical and regulatory frameworks for autonomous surgical systems remains a complex challenge, as current regulatory approaches have primarily focused on surgeon-controlled robotic platforms rather than highly autonomous technologies. Consequently, further collaboration among clinicians, engineers, regulators, and policymakers will be necessary before routine clinical implementation can be achieved.

Participants frequently described the proposed robot as a supportive technology intended to augment, rather than replace, healthcare professionals. This perspective aligns with current human–robot collaboration models, which position robotic systems as clinical support tools while preserving physician decision-making, patient communication, and individualized care. Such an approach may facilitate greater acceptance of autonomous technologies by maintaining the central role of healthcare professionals throughout clinical practice. These findings are broadly consistent with the Technology Acceptance Model (TAM), in which perceived usefulness and perceived ease of use are recognized as important determinants of technology acceptance.

Finally, participants identified several priorities for future development, including advances in artificial intelligence, real-time imaging, autonomous navigation, professional training, and comprehensive system validation. Unlike previous studies that have primarily focused on the technical capabilities of robotic surgery, participants in the present study consistently linked these priorities with physician oversight, workflow compatibility, and multidisciplinary collaboration, suggesting that successful implementation will require organizational as well as technological readiness.

Overall, this study contributes to the growing literature on healthcare technology implementation by providing multidisciplinary expert perspectives on the clinical, technical, organizational, and ethical factors that may influence the future adoption of autonomous mini surgical robots in gynecological care. Although based on an early-stage conceptual design, the findings may help inform future prototype development, implementation planning, and hypothesis generation for subsequent experimental and clinical research. The anticipated clinical benefits identified in this study should therefore be considered hypothesis-generating and implementation-oriented rather than validated clinical outcomes.

Recent clinical studies using the da Vinci SP system have suggested the feasibility and safety of next-generation single-port robotic surgery for both benign and malignant gynecological conditions [13,14,15]. Although these platforms remain surgeon-controlled rather than autonomous, they represent important milestones in the clinical translation of increasingly miniaturized robotic technologies. These findings are also consistent with the technology readiness framework proposed by Ceylan et al. (2026) [12], which argues that successful clinical translation of medical microrobots depends on demonstrating not only technical feasibility but also workflow integration, multidisciplinary collaboration, and value within existing healthcare systems.

### Limitations

This study has several limitations. First, as an exploratory pilot qualitative study based on a conceptual robotic design, the findings represent expert perceptions regarding a hypothetical system rather than evaluations of a functioning robotic device. Consequently, participants’ views may differ from those that would emerge following evaluation of an actual prototype in clinical practice. Second, although participants were purposively selected based on their professional expertise, the relatively small sample size may limit the transferability of the findings. However, no substantially new themes emerged during the later interviews, suggesting sufficient thematic richness across the overall multidisciplinary dataset within the scope of this exploratory study. Because only a small number of participants were included within each professional group, thematic saturation cannot be assumed at the subgroup level, and profession-specific findings should therefore be interpreted with appropriate caution. In addition, the participant group consisted exclusively of healthcare professionals and engineers. Patients and patient representatives were not included; therefore, the findings do not reflect end-user perspectives regarding the acceptability, trust, and usability of the proposed technology. Future studies should incorporate patient perspectives alongside expert evaluations.

Third, interviews were documented using written notes rather than audio recordings and verbatim transcription. This approach was preferred to encourage open and candid discussion, as some participants may have been less willing to express their views freely if audio-recorded. Nevertheless, although detailed notes were taken during each interview, this approach may have limited the depth and richness of the qualitative data and increased the possibility that subtle nuances in participants’ responses were not fully captured. Furthermore, because participants were asked to evaluate an emerging healthcare technology, the possibility of social desirability bias cannot be completely excluded, and some responses may have reflected favorable attitudes toward technological innovation. Future studies should include larger multidisciplinary samples, functional robot prototypes, and audio-recorded interviews with verbatim transcription to provide more comprehensive evaluations of clinical implementation and user experience.

Although participants expressed different priorities according to their professional backgrounds, no substantial disagreements regarding the overall feasibility or future potential of the proposed concept emerged during the interviews. This relatively consistent pattern may reflect the exploratory nature of the study and the fact that participants evaluated the same standardized conceptual platform with the shared objective of improving minimally invasive gynecological care. Consequently, these findings should be interpreted as preliminary expert perspectives intended to inform future prototype development and implementation planning rather than evidence supporting clinical effectiveness or routine clinical adoption.

## 5. Conclusions

The findings of this study suggest that the proposed conceptual autonomous mini surgical robot was generally perceived by multidisciplinary experts as a potentially promising concept for future minimally invasive gynecological surgery. Multidisciplinary experts perceived the potential of the proposed concept to improve procedural precision, support standardized surgical interventions, and enhance patient care, while frequently emphasizing that patient safety and physician oversight must remain central throughout clinical use.

The study further suggests that successful implementation of autonomous surgical robotics depends on more than technological advancement alone. Participants identified system validation, ethical governance, regulatory compliance, professional training, and workflow integration as key considerations for future clinical adoption. These findings provide preliminary multidisciplinary insights into expert perspectives on factors that may influence the future development and implementation of autonomous surgical technologies.

As an exploratory qualitative study based on a conceptual robotic design, the findings should be interpreted as preliminary expert perspectives rather than evidence supporting clinical effectiveness or implementation. Although based on an early-stage conceptual design, this study may help inform future prototype development, evaluation, and implementation planning for autonomous robotic technologies in women’s healthcare. However, the anticipated clinical benefits identified in this study require confirmation through prototype development, experimental testing, simulation studies, and clinical evaluation before routine clinical implementation. Future research should also include patient perspectives, larger multidisciplinary studies, and longitudinal investigations of implementation processes to better understand the feasibility, acceptability, and integration of autonomous surgical systems into clinical practice.

## Figures and Tables

**Table 1:** Summary of the themes identified through thematic analysis and perspectives across professional groups.

Participant	Profession	Academic Position	Years of Professional Experience	Relevant Expertise
P1	Gynecologist	Associate Professor	18	Antenatal care, infertility, PCOS, hysteroscopy, ultrasonography, cesarean section, genital aesthetic surgery, high-risk pregnancy
P2	Women’s Health Nurse	Assistant Professor	17	Women’s health nursing, healthcare innovation, research on robotic surgery, mobile health applications, clinical nursing management
P3	Gynecologist	Professor	27	Gynecologic oncology, laparoscopy, hysteroscopy, infertility, myomectomy, antenatal care
P4	Computer Engineer	Specialist	9	Database systems, software development, computer science
P5	Gynecologist	Professor	30	Gynecologic oncology, HPV, laparoscopy, hysteroscopy, cancer surgery, antenatal care
P6	Mechanical Engineer	Assistant Professor	20	Fluid dynamics, engineering mechanics, medical device design, thermodynamics
P7	Women’s Health Nurse	Assistant Professor	16	Women’s health nursing, patient education, web-based education, primary healthcare
P8	Mechanical Engineer	Associate Professor	15	Thermodynamics, mechatronic systems, renewable energy systems
P9	Electrical and Electronics Engineer	Associate Professor	11	Machine theory and dynamics, electronic systems, thermoelectric systems
P10	Women’s Health Nurse	Assistant Professor	16	Women’s health nursing, operating room nursing, emergency care, gender equality

**Table 2:** Themes identified through thematic analysis, representative participant statements, and primary professional perspectives.

Theme	Description	Representative Participant Statement	Primary Professional Perspective(s)
Clinical value	Perceived clinical benefits of the proposed autonomous mini surgical robot, including minimally invasive treatment and patient outcomes.	“The robot could be used for simple polyps or tubal ligation.”	Gynecologists; Women’s health nurses
Patient safety and human oversight	Importance of physician supervision, emergency interruption, and patient safety during autonomous procedures.	“The physician should confirm the procedure before irreversible actions are performed.”	Gynecologists; Anesthesiologists
Technology readiness	Technical feasibility, navigation, imaging, software reliability, and mechanical design requirements.	“Reliable autonomous navigation is essential for safe intrauterine procedures.”	Engineers
Clinical workflow integration	Integration into existing surgical practice, operating room workflow, and user training requirements.	“Comprehensive user training will be essential before clinical implementation.”	Gynecologists; Women’s health nurses
Future adoption and scalability	Future refinement, prototype validation, infrastructure, and broader implementation.	“The concept should first be validated using a functional prototype.”	Engineers; Gynecologists
Ethical governance	Ethical responsibility, accountability, legal considerations, and AI-assisted decision making.	“The physician should remain responsible for all critical clinical decisions.”	All professional groups

Note: The statements presented are representative participant statements reconstructed from contemporaneous written interview notes. They should not be interpreted as verbatim quotations because the interviews were documented using written notes rather than audio recordings and verbatim transcription. Abbreviations: AI, artificial intelligence.

## Data Availability

The data presented in this study are available from the corresponding author upon reasonable request. The data are not publicly available because they contain qualitative interview data that could compromise participant confidentiality.

## Abbreviations

The following abbreviations are used in this manuscript:

AI	Artificial Intelligence
MRI	Magnetic Resonance Imaging
